# Lipase-Catalyzed Transesterification of Egg-Yolk Phophatidylcholine with Concentrate of *n*-3 Polyunsaturated Fatty Acids from Cod Liver Oil

**DOI:** 10.3390/molecules22101771

**Published:** 2017-10-19

**Authors:** Anna Chojnacka, Witold Gładkowski, Aleksandra Grudniewska

**Affiliations:** Department of Chemistry, Wroclaw University of Environmental and Life Sciences, Norwida 25, 50-375 Wroclaw, Poland; glado@poczta.fm (W.G.); aleksandra.grudniewska@upwr.edu.pl (A.G.)

**Keywords:** PUFA-enriched phospholipids, lipases, phospholipids transesterification, *n*-3 PUFA

## Abstract

Phospholipids containing PUFAs are important vehicles for their delivering to the targeted tissues. In our research project we established enzymatic methods for the enrichment of natural egg-yolk PC with *n-3* PUFAs. Instead of synthetic PUFA ethyl esters, the new strategy was developed using polyunsaturated fatty acids enriched fraction (PUFA-EF) from cod liver oil as the natural acyl donors. PUFA-EF was produced by urea-complexation and contained 86.9% PUFA including 8.5% stearidonic acid (SDA; 18:4(*n*-3)), 26.7% EPA, and 45.2% DHA. The transesterification of PC with PUFA was catalyzed by lipases. After screening of enzymes the effect of reaction medium; molar ratio of substrates and etc. was investigated. The highest incorporation of PUFA was 45.6%; including 36.8% DHA and 5.8% EPA at the following reaction conditions: hexane; 55 °C; PUFA-EF/PC acyl ratio of 10; 48 h of reaction time and lipase B from *Candida antarctica* as a biocatalyst (20% of enzyme load).

## 1. Introduction

Docosahexaenoic acid (DHA) and eicosapentaenoic acid (EPA) belong to *n*-3 polyunsaturated fatty acids (*n*-3 PUFAs) and are also called Essential Fatty Acids (EFAs) because they can only be delivered from the diet. Both have been shown to exert profound hypolipidemic effect [1], limit hepatosteatosis [2], and display a preventative role in cardiovascular disease [3], inflammatory diseases [4], and in some cancers [5,6]. 

DHA is highly concentrated in the brain and is very important for the normal development and function of the brain [7,8]. It is also the major fatty acid in the photoreceptor membranes of the retina [9]. EPA is the precursor of prostaglandins, thromboxanes, and leukotrienes, with anti-inflammatory activity and it is also reported to have a variety of health benefits against several diseases including cancer-associated cachexia [10]. 

DHA and EPA occur in natural fish oils and micro algal oils as triacylglycerols (TAG), in krill oil as *sn*-2-PUFA phospholipids (PUFA-PL) [11], and in fish oil capsules in the form of their ethyl esters [12]. In fish oils EPA and DHA usually account for between 5% and 15% each, depending on the type of fish species, with the *n*-3 PUFA content ranging from 20 to 30% of total FA, whereas phospholipids from Antarctic krill contain 47% of the *n*-3 PUFAs in its FA profile, including 18% of DHA and 28% of EPA [13,14]. 

DHA is not synthesized in significant amounts from its precursor in human brain therefore it has to be delivered from plasma through the blood-brain barrier (BBB) [15]. Previous studies indicated that DHA-lysophosphatidylcholine (DHA-LPC) passes through the BBB about 10 times more successfully than as free fatty acids [16]. Subbaiah et al. reported that during digestion of DHA-TAG and *sn*-2-DHA phospholipids DHA is released and absorbed as free fatty acid and then re-esterified to TAG before its transport in the chylomicrons to various tissues. Compared to the *sn*-2 DHA-PC or DHA-TAG, the efficiency of DHA delivery into lymph phospholipids is five-fold higher and its incorporation into HDL is increased by two-fold, if the DHA is supplied in *sn*-1 position of dietary PC or LPC [15].

In the case of adipose tissues, it has been proven before that the major delivery road of EFAs is lipoprotein lipase-catalyzed degradation of TAG-rich lipoproteins [17] while PL are more efficient delivery form of DHA to platelets and erythrocytes than TAG [18]. *n*-3 PUFA supplements in the form of phospholipids reduce the risk of many disorders with greater efficacy than in TAG form [19,20]. In mice fed high-fat diet, it was demonstrated that *n*-3 PUFAs contained in PL are more effective than those contained in TAG in reducing hepatic steatosis, low-grade inflammation in white adipose tissue, blood lipid levels, and glycaemia [21]. Thus, PUFA-PL are effective for various applications in food and medicine.

Interest in the production of structured PLs containing specific fatty acid residues has grown significantly in recent years. Lipase-catalyzed production of PUFA-PLs is very useful because this specific reaction is carried out under mild conditions and lipases are known as an efficient tools for the preparation of TAG and glyceryl ether lipids enriched with *n*-3 PUFA [22,23]. Regioselectivity of lipases allows the specific removal or/and replacement of the acyl chains at position *sn*-1 of PL via hydrolysis and next reesterification or through direct transesterification with an acyl donor [24]. In lipase-catalyzed transesterification process commercially available fatty acid (acidolysis) [25,26] or their methyl or ethyl esters (interesterification) [27,28] are usually used as the acyl donor. The modern trend is using natural oils as a sources of triacylglycerols rich in desirable fatty acid in direct-interesterification with phospholipids [29,30]. In our research group, we elaborated the lipase-catalyzed incorporation of different biologically active acids (*n*-3 PUFA, *n*-6 PUFA, or conjugated fatty acids) into PC using natural plant oils as the acyl donors i.e., sunflower oil, linseed oil, pomegranate seed oil [31,32]. The concentrates of CLA obtained from sunflower and safflower oil were also used to produce CLA-enriched PLs [33]. 

In this work, we reported on the production of concentrate of wasted cod liver oil (CLO) and its usage as the acyl donor in the enzymatic incorporation of *n*-3 PUFA into egg-yolk PC.

## 2. Results and Discussion

The substrates for lipase-catalyzed transesterification were obtained from natural sources. PC was isolated from egg yolk and PUFA enriched fraction (PUFA-EF) was obtained from wasted CLO.

Urea complexation of saponified CLO has been chosen as a method for obtaining PUFA-EF due to its simplicity, ease of scaling and environmentally friendly procedure [34]. The fatty acid compositions of the original CLO and the PUFA fraction obtained after urea complexation is given in Table 1.

Starting from 25 g of CLO 3.9 g of PUFA-EF as a mixture of free fatty acids were obtained. CLO contained 27.1% of *n*-3 PUFA with DHA and EPA constituting about 10% and 11%, respectively. The urea crystallization method allowed to enrich the *n*-3 PUFA fraction with a high selectivity towards DHA, which in the PUFA-EF was the major fatty acid followed by EPA and SDA. The total amount of *n*-3 PUFA increased more than three times after crystallization process and in PUFA-EF it reached about 87%. Almost four-fold increase of DHA, 2.5-fold increase of EPA and three-fold increase of SDA was observed (Table 1).

The fatty acid composition of native PC obtained from egg-yolk is also shown in Table 1. The results indicate that palmitic acid (C16:0) and oleic acid (C18:1) are the predominant fatty acids and each of them makes up a third of the total. The next most abundant fatty acids in yolk-PC are stearic (C18:0; 15.7%) and linoleic (C18:2; 15.3%). The content of polyunsaturated fatty acids, especially from the *n*-3 family is relatively low (in this case does not exceed 4%), therefore enrichment of egg-yolk PC with *n*-3 PUFA is more justified from a nutritional point of view compared with soy PC. It is known that when hen feed is supplemented with some plant or fish oils the enrichment of egg-yolk lipids (especially PC) with *n*-3 PUFA is observed, however the amount of these acids in PC fraction usually does not exceed 10% of total fatty acids [35,36,37]. Additionally, deposition of DHA and EPA is predominantly observed at *sn*-2 position of phospholipids [35].

### 2.1. Screening of Enzymes

**Scheme 1:**
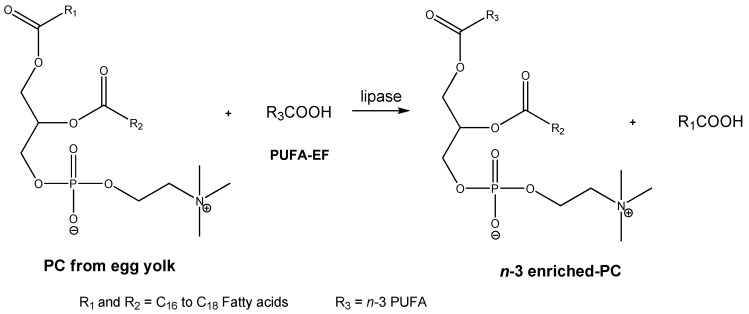
Lipase-catalyzed acidolysis of egg-yolk PC with PUFA-EF.

In this study, seven different lipase preparations were examined for their ability to catalyze the acidolysis process between egg-yolk PC and PUFA-EF (Scheme 1).

Five preparations contain different lipases: Lipozyme TL IM (a silica granulated *Thermomyces lanuginosus* lipase preparation), CALA (lipase A from *Candida antarctica* immobilized on resin Immobead 150), Lipozyme^®^ (lipase from *Mucor miehei* immobilized on an anion exchange resin), Amano PS IM (lipase from *Burkholderia cepacia* immobilized on diatomaceous earth) and non-immobilized lipase from *Candida cylindracea*. The other two preparations contain the same lipase (lipase B from *Candida antarctica)* but immobilized on different carriers: Novozym 435 (immobilized on a macroporous acrylic resin) and CALB (immobilized on resin Immobead 150). Although the enzymes exhibited different activities (according to suppliers), we decided to apply them at the same weight ratio, because it is important to reduce the total costs of a process while choosing an enzyme for industrial applications. Initial conditions applied for the reaction systems in our present investigations were also chosen on the basis of earlier studies [26]. They were as follows: 20% lipase dosage (based on the weight of substrates), temperature 55 °C, PC/PUFA-EF molar ratio 1/3 and hexane as a solvent.

**Figure 1:**
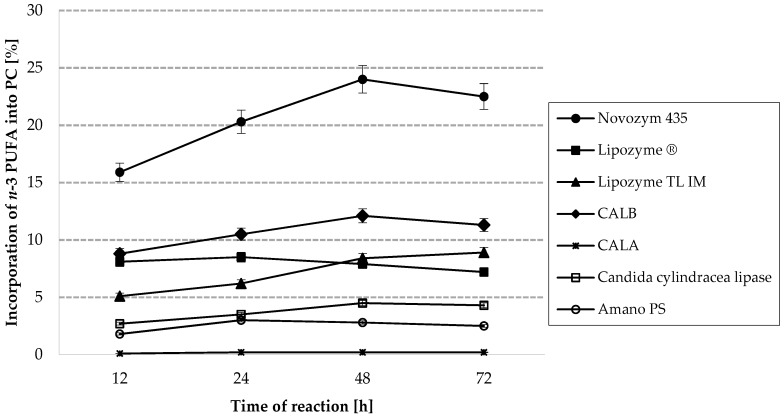
Time courses of different lipases-catalyzed acidolysis between egg-yolk PC and PUFA-EF. Reaction condition: temperature, 55 °C; PC/PUFA-EF molar ratio, 1/3; lipase dosage, 20%; solvent, hexane.

The time course of the incorporation of *n*-3 PUFA into PC by lipases is shown in Figure 1.

Only two preparations of lipase B from *Candida antarctica* exhibited satisfactory activity, giving more than 10% incorporation of *n*-3 PUFA into PC within 48 h. In the reaction with Novozym 435 the incorporation degree reached a maximum (23.7%) after 48 h, whereas CALB-catalyzed acidolysis afforded the double-less incorporation degree (12.1%) at the same time. When the reaction was continued for more than 72 h no significant increase of *n*-3 PUFA in modified PC was observed. For other enzymes the degree of incorporation decreased in the following order after 48 h of reaction: Lipozyme TL IM ≅ Lipozyme^®^ > *C. cylindracea* lipase > Amano PS. Lipase A from *Candida antarctica* (CALA) was almost inactive in acidolysis process giving less than 0.5% of *n*-3 PUFA incorporation. 

Our screening results were in accordance with the results reported by Lyberg et al. They achieved the best incorporation of *n*-3 PUFA in esterification of 2-palmitoyl-LPC using *Candida antarctica* lipase B [38].

Because the highest degree of incorporation of *n*-3 PUFA into PC was achieved for Novozym 435, this enzyme was selected for subsequent experiments.

### 2.2. Effect of Substrate Molar Ratio

**Figure 2:**
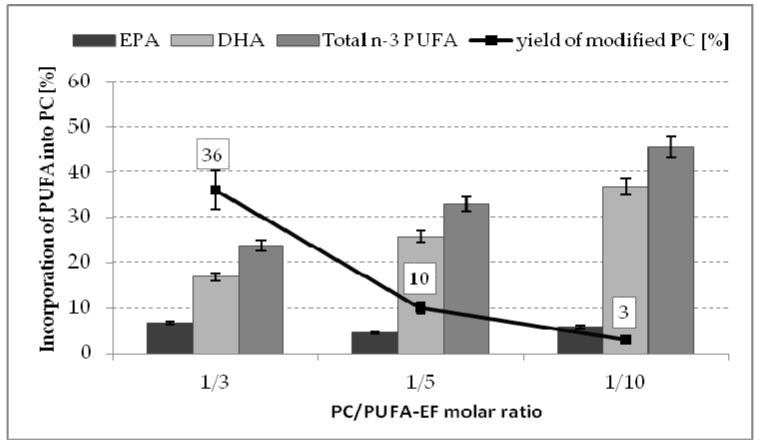
Effect of substrate molar ratio (PC/PUFA-EF) on the incorporation of *n*-3 PUFA into egg-yolk PC in acidolysis reaction catalyzed by Novozym 435 (reaction condition: enzyme load 20% (*w*/*w*), temperature 55 °C, solvent hexane, reaction time 48 h).

The increase of substrate molar ratio (PC/PUFA-EF) from 1/3 to 1/10 resulted in higher incorporation of *n*-3 PUFA, which grew up from 23.7 to 45.5% (Figure 2). An over two-fold increase was also observed for incorporation of DHA, which content in modified PC made up 39.8% of total fatty acids after reaction carried out at 1/10, PC/PUFA-EF molar ratio. On the other hand, the content of EPA was not significantly different, irrespective of molar ratio used. It can be explained by higher specificity of enzyme used (Novozym 435) towards DHA than EPA. Peng et al. observed reversed specificity towards these acids using Lipozyme TL IM [25].

A significant decrease of the isolated yield was observed for modified PC along with the increase of PUFA-EF in the reaction mixture. The highest isolated yield (36%) was obtained at 1/3 substrate molar ratio, significantly lower value (3%) was noticed in the case of 1/10 ratio. 

Adlercreutz et al. observed that the increase in FA concentration led to increase of PC yield [39] while Vikbjerg et al. reported no significant effect of PC/FA molar ratio on yield of modified PC [40]. However in both investigations the amounts of PC and LPC were measured by HPLC or TLC-FID methods. Taking into consideration all steps of modified PC preparation including purification of the product by column chromatography we estimated the isolated yield of PC as the amount of recovered PC (by weight) in relation to initial PC. In this context the lower yield of modified PC observed at highest PUFA-EF concentration can be explained by difficult separation of the products from the reaction mixtures.

Taking into account the economy of the process, 1/3 molar ratio (PC/PUFA-EF) was chosen for subsequent experiments. 

### 2.3. The Effect of Organic Solvent

**Figure 3:**
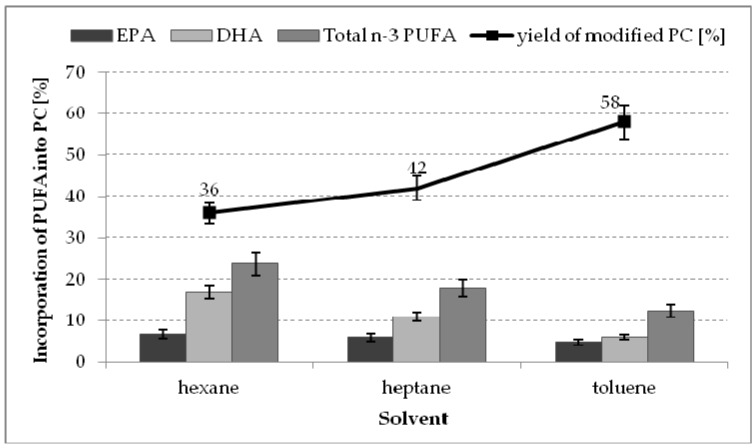
Effect of organic solvent on the incorporation of *n*-3 PUFA into egg-yolk PC in acidolysis reaction catalyzed by Novozym 435 (reaction condition: enzyme load 20% (*w*/*w*); temperature 55 °C; PC/PUFA-EF molar ratio 1/3; reaction time 48 h).

The best results of incorporation degree were obtained in hexane (Figure 3). Using heptane as a solvent resulted in a significant decrease of *n*-3 PUFA incorporation from 24 to 18% and DHA incorporation was lowered by 6%. The content of EPA was not changed significantly and leveled off at 6.6%. The lowest incorporation of *n*-3 PUFA (12.3%), DHA (6%), and EPA (4.8%) was observed in toluene. Mutua and Akoh also achieved the highest incorporation of *n*-3 PUFA into PC in hexane using nonimmobilized lipase from *Mucor miehei* [41]. Simultaneously, the relationship between low incorporation level and high isolated yield of modified PC was observed and for toluene the 58% yield was obtained whereas in hexane 36% was achieved. 

### 2.4. The Effect of Enzyme Dosage

**Figure 4:**
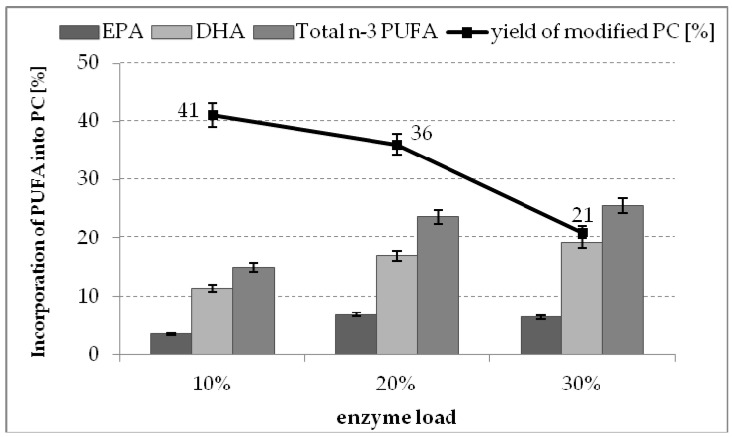
Effect of enzyme load on the incorporation of *n*-3 PUFA into egg-yolk PC in acidolysis reaction catalyzed by Novozym 435 (reaction condition: temperature 55 °C; PC/PUFA-EF molar ratio 1/3; solvent hexane; reaction time 48 h).

The increase of enzyme dosage from 10 to 20% resulted in significant increase of incorporation of *n*-3 PUFA, from 15 to 24% (Figure 4). The incorporation of DHA and EPA was also higher, by 5.5% and 3%, respectively, but at the same time the isolated yield of modified PC was lowered from 41 to 36%. Increase of enzyme load to 30% did not affect incorporation degree but a further decrease of isolated yield was observed. Taking into consideration the cost of enzyme, 20% of enzyme load seems to be enough to achieve both high incorporation of PUFA and PC yield.

### 2.5. Positional Analysis of Modified PC

The products of reaction obtained in the Novozym 435-catalyzed reaction (conditions: temperature 55 °C; PC/PUFA-EF molar ratio 1/3; 20% enzyme load; solvent hexane; reaction time 48 h) were separated by column chromatography to afford 36.2% modified PC and 48% LPC. LPC was formed as a product of partial hydrolysis of the PC during the acidolysis reaction and it was found to contain over 22% of *n*-3 PUFA. Analysis of positional distribution of fatty acids in egg-yolk PC before and after modification (Table 2) indicated that *n*-3 PUFA were almost exclusively incorporated into the *sn*-1 position of the glycerol skeleton and their content in this position reached 47.4%. Analyzing the total and positional FA composition of the native and modified PC and LPC one can see the increase of PUFA was accompanied by a reduction in saturated fatty acids which usually occupy the *sn*-1 position of egg-yolk PC. In the case of both (palmitic; C16:0, and stearic; C18:0) acids, almost two-fold decrease in their content was observed. These data confirm the specificity of Novozym 435 lipase towards *sn*-1 position of PC. Observed regioselectivity is not a general rule for other lipases; Yamamoto et al. used lipase OF from *Candida rugosa* to incorporate *n*-3 PUFA into *sn*-2 position of soy PC [42].

Enzymatic preparation of *n*-3 PUFA-PL using fish oils or their concentrates as the acyl donors is a subject of interest to different research groups. However, most of them concern the modification of soy PC [25,42,43,44,45,46]. Using phospholipase A_2_ in enzymatic modification is a limitation because only *sn*-2 PUFA-PC can be obtained and requires a two-step procedure [47,48].

Introduction of *n*-3 PUFA into *sn*-1 position of PC can be obtained using phospholipase A_1_ but low incorporation is observed for free enzyme (22%) [44] and immobilization of enzyme on different carrier is necessary to achieve satisfactory content of *n*-3 PUFA in PC [45,46]. Xi et al. applied harsh reaction conditions (12 MPa, 50 °C, supercritical CO_2_ as a solvent) to increase DHA in Antarctic krill PC from 15 to 59% [49].

The alternative for phospholipase A_1_ in modification of *sn*-1 position of PC are commercially available lipases with selectivity towards *sn*-1 position. Totani and Hara carried out interesterification between soy-PC and sardine oil using lipases from *Candida cylindracea* and *Rhizopus delemar* [43]. Higher incorporation of *n*-3 PUFA into PC (17.6%) was obtained in *C. cylindracea*-catalyzed reaction. Comparable results (18.9% incorporation) were reported by Peng et al. in the Lipozyme TL IM-catalyzed acidolysis of soy-PC with *n*-3 PUFA concentrate after 72 h of reaction [25].

To the best of our knowledge, the only results on lipase-catalyzed modification of egg yolk PC using commercial concentrate of fish oil were published by Haraldsson and Thorarensen [50]. They used 1,3-regiospecific *Rhizomucor miehei* lipase (Lipozyme™) in the solvent free acidolysis reaction and phospholipids with 48% *n*-3 PUFA were obtained presumably as a mixture of PC and LPC. The highest incorporation degree obtained in our investigation (45.6%) concern only PC. Using concentrate with different FA composition (45.2% DHA and 26.7% EPA in our investigation versus 55% EPA and 30% DHA in studies of Haraldsson and Thorarensen [50]) resulted in higher content of DHA in obtained product (36.8% in modified PC versus 16% in modified PL). It is also worth to notice that in the cited studies [50], 100% dosage of lipase and longer reaction time (72 h) were applied to achieve such incorporation whereas our conditions involve maximum 30% enzyme load and 48 h of reaction.

## 3. Materials and Methods

### 3.1. Materials and Chemicals

Lohmann Brown hens’ eggs were a gift from the Tronina factory. Cod liver oil (CLO) was a gift from the National Marine Fisheries Research Institute, Gdynia, Poland. Lipozyme TL IM (a silica granulated *Thermomyces lanuginosus* lipase preparation, 250 U/g) was a gift from the Novozymes A/S (Bagsvaerd, Denmark). Lipase B from *Candida antarctica* immobilized in a macroporous acrylic resin (synonym: Novozym 435, >5000 U/g), lipase B from *Candida antarctica* (CALB, >1800 U/g), lipase A from *Candida antarctica* (CALA, >500 U/g) both immobilized on resin Immobead 150, lipase from *Mucor miehei* (Lipozyme^®^, >30 U/g), lipase from *Candida cylindracea* (≥2 U/mg) and lipase from *Burkholderia cepacia* (Amano PS IM, >500 U/g) were purchased from Sigma-Aldrich (St. Louis, MO, USA). A boron trifluoride methanol complex solution (13–15% BF_3_ × MeOH) was purchased from Sigma-Aldrich (St. Louis, MO, USA). Silica gel-coated aluminum plates (Kieselgel 60 F254, 0.2 mm) used in thin layer chromatography (TLC) and the silica gel (Kieselgel 60, 230–400 mesh) used in the column chromatography were purchased from Merck.

### 3.2. Isolation of PC from Egg Yolk

The extraction of phospholipids from egg yolk was performed on a semi-technical scale in Wroclaw Technology Park. Eggs were purchased from the poultry farm “Ovopol” (Nowa Sól, Poland) and dried in the drying chamber at inlet air temperature 185 ± 5 °C and an outlet air temperature 70 ± 2 °C. In the next step, obtained powder was extracted with ethanol in a tank equipped with a mechanical stir maintaining the dilution ratio of yolk to solvent at 1:4 (*m*/*v*). The process of suspension was carried out for 90 min. and then alcohol was removed by filtration. The residue was evaporated in vacuo (0.06 MPa at 50 °C). The pure PC was separated from crude PLs fraction by silica gel column chromatography (chloroform/methanol/water, 65:25:4, *v*/*v*/*v*). Purity of PC fractions was analyzed by TLC on silica gel-coated aluminum plates (chloroform/methanol/water, 65:25:4, *v*/*v*/*v*) and HPLC (Section 3.5). Pure phosphatidylcholine fractions were collected and evaporated in vacuo.

### 3.3. Production of n-3 PUFA Concentrate

The raw material for the production of the concentrate was cod liver oil containing approximately 30% PUFA. Concentration was carried out by a two-step process: saponification of triacylglycerols of CLO followed by formation of urea inclusion compounds according to the procedure described by Patkowska-Sokoła et al. [51]. 

The mixture of 25 g of CLO and 75 mL 1 M NaOH ethanol solution was heated under reflux for 1 h. Then the mixture was cooled to room temperature, 100 mL distilled water was added and such mixture was extracted with hexane. Organic layer containing unsaponifiable residue was rejected and the water layer was acidified to pH 1 with 6 M HCl, extracted with hexane, and dried under anhydrous MgSO_4._ The hexane was then evaporated in vacuo.

To 20 g of free fatty acids obtained after saponification of CLO, 80 g of urea and 150 mL of methanol was added. The mixture was stirred for 1 h on a magnetic stirrer at 60 °C. The solutions were allowed to crystallize in a refrigerator at 4 °C for 18 h. The crystal fraction was filtrated on a Büchner funnel. The filtrate was diluted with 15 mL of distilled water and acidified to pH 4–5 with 3 M HCl. An equivalent quantity of hexane was added to the solution and the fatty acids were extracted twice. The hexane fractions were washed with distilled water to remove leftover of urea and dried over anhydrous magnesium sulfate. Hexane was evaporated to obtain the PUFA-enriched fraction (PUFA-EF). The fatty acid compositions of this fraction was analyzed by GC (Table 1).

### 3.4. The Lipase-Catalyzed Transesterifiction of PC with PUFA Concentrates

The egg-yolk PC (0.13 mmol, 100 mg) was mixed with PUFA-EF at molar ratio of substrates 1/3 (PC/PUFA-EF) in 5 mL of solvent and then 20% of lipase (by weight of substrates) was added. The reactions were carried out using seven different lipases, in N_2_ atmosphere, at 55 °C. The effect of molar ratio of substrates, lipase dosage and different solvent was tested in another set of experiments for Novozym 435. The reaction mixtures were agitated in a magnetic stirrer at 300 rpm and stopped at the selected time intervals by enzyme filtration. Modified PC and lysophosphatidylcholine (LPC) were separated from the mixtures by silica-gel column chromatography (chloroform/methanol/water, 65:25:4, *v*/*v*/*v*). All experiments were carried out in triplicates.

### 3.5. Analysis of Substrates and Products

Purity of native and modified egg-yolk PC was analyzed by HPLC on an Ultimate 3000 DIONEX chromatograph equipped with Corona^TM^ Charged Aerosol Detector (CAD). A Waters Spherisorb S5W column (150 × 4.6 mm) was used for analysis. HPLC gradient program was as follows: (flow rate 0.6 mL × min^−1^): 0 min 0/90/10 (%A/%B/%C) at 2 min, 0/40/60 at 20 min, 1/40/59 at 22 min, 10/40/50 at 38 min, 8/40/52 at 44 min, 1/40/59 at 55 min, 0/90/10 at 56 min, 0/90/10 hold 10 min (A/B/C, water/0.1% solution of formic acid in hexane/isopropanol).

Fatty acid profiles of starting materials and products were determined after their conversion to the corresponding fatty acid methyl esters (FAME) according to the following procedure: samples (50 mg) were heating under reflux (3 min) with 3 mL of BF_3_×MeOH complex solution and then cooled. Products were extracted with 2 mL of hexane and the organic layers were washed with a saturated NaCl solution. Hexane extracts were dried and analyzed directly by gas chromatography (GC) on an Agilent 6890 N instrument equipped with a 70% cyanopropyl polysilphenylene-siloxane column (TR FAME, 30 m length, 0.25 mm diameter, 0.25 μm film thickness). The oven temperature was first set at 160 °C for 3 min and then raised to 220 °C (rate 5 °C min^−1^) and next to 260 °C at 30 °C min^−1^ and held there for 3 min. The injector and flame ionization detector temperatures were 250 °C and 280 °C, respectively. The FAME were identified by comparing their retention times with those of a standard FAME mixture (Supelco 37 FAME Mix) purchased from Sigma-Aldrich. The incorporation degree of *n*-3 PUFA into PC was calculated as follows:

Incorporation of *n*-3 PUFA into PC = % of *n*-3 PUFA in modified PC − % of *n*-3 PUFA in native PC

Similar equation was applied for calculation of incorporation of particular fatty acid.

### 3.6. Positional Analysis of Fatty Acids in Native and Modified PC

The procedure was based on regiospecific Lipozyme^®^-catalyzed ethanolysis of PC. The details of the procedure were described in our previous paper [52].

## 4. Conclusions

Summarizing, the developed method of enzymatic preparation of *n*-3 PUFA enriched-PL reported herein is competitive with those reported previously. This method involves mild reaction conditions, utilization of natural substrates (egg-yolk PC and waste cod liver oil) and commercially available regioselective enzyme (Novozym 435). As a result, molecular species i.e., *sn*-1 DHA enriched-PC and DHA enriched-LPC as carriers of this biologically active molecule overcoming blood-brain barrier (BBB) can be easily obtained. This molecular species can target the brain more effectively than DHA-TAG as was reported earlier in the in vivo studies of Kitson et al. concerning brain uptake of DHA-PC with radiolabelled tracers in rats [53].

## Figures and Tables

**Table 1:** Fatty acid residue composition (wt. %) of cod liver oil (CLO) and the polyunsaturated fatty acids enriched fraction (PUFA-EF) after urea crystallization. ^a^

Fatty Acid	CLO	PUFA-EF	Native PC
C16:0	11.0 ± 0.64	1.0 ± 0.16	34.2 ± 0.11
C18:0	2.3 ± 0.26	0.1 ± 0.04	15.7 ± 0.05
C18:1	19.5 ± 0.88	3.9 ± 0.11	29.0 ± 0.04
C18:2 *n*-6	1.6 ± 0.04	1.2 ± 0.03	15.3 ± 0.02
C18:3 *n*-3	0.8 ± 0.03	2.3 ± 0.07	-
C18:4 SDA *n*-3	2.7 ± 0.11	8.5 ± 0.45	-
C20:4 *n*-6	0.9 ± 0.01	2.2 ± 0.05	2.0 ± 0.01
C20:5 EPA *n*-3	10.4 ± 0.04	26.7 ± 0.11	0.6 ± 0.02
C21:5 *n*-3	0.5 ± 0.02	1.7 ± 0.02	-
C22:5 *n*-3	1.5 ± 0.04	2.5 ± 0.01	-
C22:6 (DHA) *n*-3	11.2 ± 0.13	45.2 ± 0.35	3.2 ± 0.05
Others	37.6	4.7	-
Total *n*-3 PUFA	27.1	86.9	3.8

^a^ Data show mean ± SD with *n* = 3.

**Table 2:** Fatty acid composition (wt %) of native egg yolk PC and modified PC and lyso-PC. ^a^

Fatty Acid	Native PC			Modified PC			Modified LPC
	Total	*sn*-1	*sn*-2	Total	*sn*-1	*sn*-2	
C16:0	34.2 ± 0.11	64.1 ± 0.22	0.8 ± 0.04	15.7 ± 0.48	28.5 ± 0.65	0.5 ± 0.55	17.7 ± 0.22
C18:0	15.7 ± 0.05	29.2 ± 0.55	1.1 ± 0.09	9.5 ± 0.27	19.5 ± 0.35	0.3 ± 0.13	10.8 ± 0.05
C18:1	29.0 ± 0.04	5.5 ± 0.33	56.3 ± 0.32	30.1 ± 0.09	7.5 ± 0.12	52.1 ± 0.95	28.1 ± 0.56
C18:2	15.3 ± 0.02	1.2 ± 0.05	28.8 ± 0.55	15.9 ± 0.34	1.5 ± 0.25	29.0 ± 0.84	20.5 ± 0.08
C18:4 SDA *n*-3	-	-	-	2.1 ± 0.06	3.8 ± 0.22	0.2 ± 0.06	1.9 ± 0.02
C20:4 *n*-6	2.0 ± 0.01	-	4.5 ± 0.06	1.8 ± 0.08	3.5 ± 0.12	0.5 ± 0.08	0.7 ± 0.03
C20:5 EPA *n*-3	0.6 ± 0.02	-	1.4 ± 0.06	7.8 ± 0.13	14.5 ± 0.35	1.5 ± 0.12	5.9 ± 0.05
C22:6 (DHA) *n*-3	3.2 ± 0.05	-	7.1 ± 0.22	17.6 ± 0.11	29.1 ± 0.84	3.2 ± 0.55	14.4 ± 0.07
Total *n*-3 PUFA	3.8	0	8.5	27.5	47.4	4.9	22.2

^a^ Data show mean ± SD with *n* = 3.
